# A comparative review of artificial muscles for microsystem applications

**DOI:** 10.1038/s41378-021-00323-5

**Published:** 2021-11-23

**Authors:** Mayue Shi, Eric M. Yeatman

**Affiliations:** Department of Electrical and Electronic Engineering, Imperial College London, Exhibition Road, London, SW7 2AZ UK

**Keywords:** Engineering, Materials science

## Abstract

Artificial muscles are capable of generating actuation in microsystems with outstanding compliance. Recent years have witnessed a growing academic interest in artificial muscles and their application in many areas, such as soft robotics and biomedical devices. This paper aims to provide a comparative review of recent advances in artificial muscle based on various operating mechanisms. The advantages and limitations of each operating mechanism are analyzed and compared. According to the unique application requirements and electrical and mechanical properties of the muscle types, we suggest suitable artificial muscle mechanisms for specific microsystem applications. Finally, we discuss potential strategies for energy delivery, conversion, and storage to promote the energy autonomy of microrobotic systems at a system level.

## Introduction

With the miniaturization of electronics and the development of machine intelligence, various advanced electronic devices have been increasingly deployed in a wide range of living environments in the last few decades. Frequent and close human-machine interactions require devices that are soft, small-scale, lightweight, and power-efficient. Actuators, as a critical part of many electronic devices, also require these properties. However, conventional rigid actuators, such as electric motors and combustion engines, not only have safety problems but also suffer low overall efficiency and high cost^1^ on the subcentimeter scale because of the mechanical impedance mismatch, significant friction loss, and complexity of fabrication.

Natural muscle is a soft biological actuator with outstanding driving capability, compliance, and additional functions such as self-healing. Specifically, skeletal muscles are composed of muscle fibers that are arranged in parallel between tendons, while muscle fibers are composed of myofibrils. A myofibril contains myosin filaments and actin filaments. The myofibril contracts when the actin filaments slide along the myosin filaments, energized by the hydrolysis of ATP (adenosine triphosphate). This process is under the control of neurons through the release of Ca^2+^. A relaxation process occurs due to the cessation of the interaction between actin and myosin when the concentration of Ca^2+^ decreases. The movement is finally transferred to bones through tendons at the ends of the skeletal muscles. The detailed structure and molecular basis of skeletal muscles have been extensively explored, as shown in the existing literature^2^.

The excellent performance of natural muscles inspires researchers to realize such attractive functions with engineering materials and methods. Artificial muscles are a category of rapidly developing actuators that can mimic the properties and functions of natural muscle and are suitable for compliant interactions. G.M. Whitesides defined an actuator as a device that supplies mechanical energy to another device^3^. Based on this definition, the artificial muscle is clearly an actuator; however, it has several critical differences from conventional actuators, such as electric motors and pneumatic pistons, which have been widely used in industry. S.M. Mirvakili and I.W. Hunter defined that artificial muscles are a class of responsive materials and devices that can reversibly generate actuation, including contraction, expansion, or rotation within one component^4^. In our view, an artificial muscle has three typical features: small scale, low stiffness, and the capability of replicating a central function of natural muscle, i.e., generating actuation within an individual small device in response to an external stimulus. This stimulus can take many forms^4^, including an electrical signal^5^, pressure^3^, temperature^6^, a magnetic field^7,8^, etc.

Artificial muscles are particularly suitable for human-machine interaction applications because of their soft and tough mechanical properties, flexible drive mechanisms, low fabrication complexity, and acceptable efficiency at a miniature (subcentimeter) scale. In recent years, artificial muscles have developed rapidly, driven in part by new and improved materials, fabrication processes, and device structures. There has been an increasing amount of literature on artificial muscles based on a variety of actuation mechanisms, such as small-scale pneumatic/hydraulic artificial muscles^3,8^, dielectric elastomer (DE) artificial muscles^9^, ionic polymeric actuators^10^, micro piezoelectric actuators^11^, magnetic actuators^7^, and actuators based on shape memory alloys and polymers (SMAs and SMPs)^12^. A number of excellent reviews of various artificial muscles have been published in recent years. For example, Mirvakili et al.^4^ reviewed various actuation mechanisms for artificial muscles and their mechanical properties. Hines et al.^13^ summarized and discussed a variety of soft actuators categorized by different driving stimuli.

**Fig. 1:**
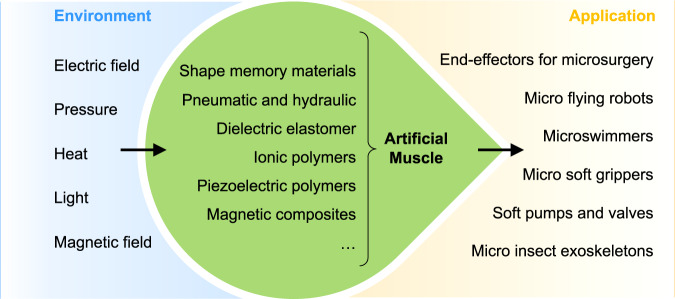
Artificial muscles for microsystem applications. Artificial muscles are responsive to various environmental stimuli. They can generate actuation with high compliance for different microsystem applications

This study aims to provide a comparative review with practical opinions on the application and development of artificial muscles in microsystems based on previous reviews and some critical developments more recently. Specifically, the first aim of this paper is to review recent research into artificial muscles. We provide an overview of artificial muscles based on six important actuation principles, as shown in Fig. 1, and especially focus on the progress over the past five years. Second, in this review, we both summarize the properties of various actuation mechanisms and emphasize a systematic comparative analysis of electrical and mechanical properties. Parameters significant for potential applications are highlighted, such as modulus, maximum stroke, response time, and power efficiency. The pros and cons of different kinds of artificial muscle are analyzed and compared. According to these analyses, we suggest suitable artificial muscle types for specific applications based on their unique electrical and mechanical properties. Finally, to realize highly efficient energy management, we discuss some methods involving energy delivery, storage, and conversion. This is especially useful for pursuing energy autonomy in microrobotic systems.

## Artificial muscles with various actuating mechanisms

Many stimuli and mechanisms that drive artificial muscles have been investigated in recent years. According to different stimuli, artificial muscles can be categorized as thermoresponsive^6,14^, electrically responsive^15–17^, magnetically responsive^7,18,19^, photoresponsive^20^, chemically responsive^21^, and pressure driven^22,23^. Furthermore, some artificial muscles are multistimuli responsive^24^. The multiresponsive mechanism effectively increases the flexibility and adaptability of an artificial muscle but requires complex control of the device and ambient environment. According to specific actuating mechanisms, artificial muscles can be classified as SMAs and SMPs, DEs, ionic polymer-metal composites (IPMCs), pneumatic, hydraulic actuators, etc. This classification method is preferred in our review because it emphasizes the physical essence applied to generate actuation and thus can provide clear guidance for technical improvement. In this section, we aim to summarize properties and recent progress in six common actuating mechanisms.

### SMAs and SMPs

The shape memory effect describes a phenomenon in which a material can be deformed and fixed into a temporary shape, after which recovery of the original shape can be realized by an external stimulus^25^. There are one-way and reversible (two-way) shape memory effects; some shape memory materials even have multishape memory properties^26^. The one-way shape memory effect allows one-way recovery from the temporary shape, while the reversible effect allows the material to be deformed between two shapes. The shape memory effect has been found in some alloys^27,28^, ceramics^29,30^, and polymers^25,31,32^. Among them, SMAs have been studied extensively since the first discovery in 1932^33^. W. Buehler and F. Wang discovered and developed the famous NiTi-based SMA (nitinol) in 1962. At present, NiTi-based SMAs are still the most preferable in applications compared to other SMAs, such as iron-based and copper-based SMAs^34^. Although iron-based and copper-based SMAs are low-cost and commercially available, their poor stability and thermomechanical performance often limit their further application^4,27^.

The shape memory effect in alloys originates from a reversible phase transition when the temperature varies. In the process of a phase transition, the SMA has three different crystal structures: twinned martensite, detwinned martensite, and austenite^27,35^. A typical one-way shape memory process can start from the twinned martensite at low temperatures. With mechanical loading, the twinned martensite transforms to detwinned martensite with deformation (deformed martensitic phase). Then, when the deformed SMA is heated to a temperature higher than the austenite start temperature (*A*_*s*_), it gradually recovers to its original shape and transforms to austenite. In this step, conductive SMAs can be easily driven with Joule heat. Finally, after a cooling process, the austenite returns to the twinned martensitic phase with the original shape.

The most significant limitation for SMAs is their low working frequency, which is caused by the low heat transfer rate, especially in the cooling stage. Several strategies have been explored to enhance heat transfer. For example, reducing the diameter of SMA fibers has proven beneficial due to an increase in the specific surface area^36^. Interestingly, this suggests that a microactuator based on an SMA could perform at a higher operating frequency than mesoscale devices. Moreover, heat convection has been studied to increase the response speed. An et al. provided a summary of relevant methods in ref. ^27^.

In recent years, the soft nature of SMPs has attracted increasing attention. SMPs are particularly promising as artificial muscles because they show similar properties in several aspects to real muscle, namely, low mass density, low elastic modulus, and high shape recovery rate. Moreover, SMPs such as polylactic acid and polycaprolactone are biocompatible and biodegradable, which makes them particularly suitable for biomedical applications. The recovery time is often on the level of 1–100 s, and a typical produced force ranges from 1 to 100 MPa.

A one-way SMP can be first deformed at an elevated deformation temperature (*T*_*d*_), after which the deformed shape can be fixed with shape fixity (*R*_*f*_) while cooling. This temporary deformation will be released with shape recovery (*R*_*r*_) by heating the material to a recovery temperature (*T*_*r*_)^26^. For a reversible SMP, the programming process is similar. In ref. ^25^, Lendlein and Gould provided a detailed discussion of the macroscale and molecular-scale mechanisms of an SMP. Briefly, the chain segments in the polymer are crosslinked through netpoints chemically and physically. These netpoints control the permanent shape at the macroscale. The temporary shape is determined by the additional reversible netpoints, which are formed during the programming process. These factors limit the recovery of oriented chain segments after programming, therefore fixing the temporary shape. If there is more than one phase transition temperature for different domains, then the SMP will have multiple shape memory effects^37,38^. A reversible shape memory effect is critical for soft actuators, but most conventional SMPs are one-way shape memory materials. The first reversible bidirectional SMP actuator was developed by Behl et al.^39,40^. In contrast to a one-way SMP, which has a “frozen” state at low temperature, deformation in the direction of orientation occurs in a reversible SMP during the cooling process due to a crystallization-induced elongation of the oriented segments associated with the lower melting temperature^40^.

**Fig. 2:**
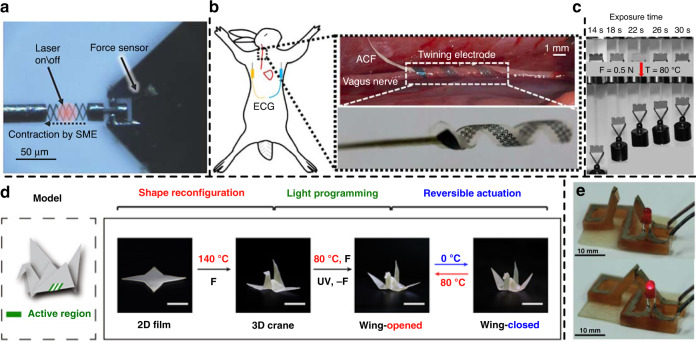
SMA- and SMP-based microactuating structures and devices. **a** SMA microactuator with diamond-shaped frame structures (1 µm in thickness) in the laser irradiation test^44^. Copyright 2018 by Wiley. Reprinted with permission. **b** Schematic diagram of twining electrodes using SMP for vagus nerve stimulation and recording of ECG (left), and photos of an implanted twining electrode (inner diameter of 1 mm) on the vagus nerve (right)^49^. Reproduced from ref. ^49^ (2019, CC BY 4.0). **c** 4D printed SMP with variable modulus^52^. Copyright 2019 by ACS. Reprinted with permission. **d** Programming of an artificial crane using thermo and photoreversible SMP^50^. Reproduced from ref. ^50^ (2018, CC BY 4.0). **e** 3D printed SMP switch with silver conductive ink^51^. Copyright 2016 by Wiley. Reprinted with permission

Shape memory artificial muscles have been used for various applications^41,42^. Kim et al. used a NiTi spring as the muscle of an earthworm-like soft robot^43^. The spring structure was fabricated by winding the NiTi fiber (100 μm) around a core wire (200 μm). The spring-shaped muscle fibers are arranged in two directions to mimic the longitudinal muscle and circumferential muscle of the earthworm. In a further experiment, the robot exhibited a 50% contraction and an energy density of 1226 J/kg. Lee et al. developed microscale NiTi actuators with a diamond-shaped frame structure fabricated by focused ion beam (FIB) milling^44^ (Fig. 2a). When driven by laser heating, these actuators demonstrated a maximum strain of 60%, which was much higher than that of conventional bulk NiTi alloy (strain < 8%^27^). Notably, these actuators are able to operate at high frequencies. Because of its extremely small volume, their device had a low thermal capacitance and a high specific surface area, which led to a very fast thermomechanical response up to 1.6 kHz.

Because of the transparency of some SMPs, they have been studied as optical components in recent years. For example, an SMP light valve array was developed by Xu et al.^45^. The microstructured SMP was formed by single-step compression molding against a polydimethylsiloxane (PDMS) mold and then compressed against a flat mold at ~100 °C to form a temporary flat shape with a transmittance of ~50%. When heated by an indium tin oxide array with Joule heat on a transparent substrate, the microstructure could be selectively recovered, and the transmittance decreased to less than 0.8% after recovery. Another study reported recently by Zhang et al. shows that SMPs with submicrometer structures had more possibilities in displaying images with colors^46^.

SMPs can also be applied to realize the controllable deformation of structures. Complex geometries and locking assemblies with sequential folds have been realized using SMPs^47,48^. Figure 2b presents a biomedical application of the controllable deformation^49^. The SMP was used to form a twining electrode to realize vagus nerve stimulation and a recording of ECG. Figure 2d presents the programming and operating process of an artificial crane^50^ with an SMP, which is programmable with thermo and photoreversible bonds. The wings showed reversible actuation while the temperature varied between 0 and 80 °C. 3D printing has been a flexible method for the fabrication of SMP actuators. Figure 2e presents a 3D printed SMP switch with silver conductive ink^51^. This actuator can switch between on and off states at different temperatures. Figure 2c presents 4D printed SMPs (there is an additional dimension of time) with different moduli and a hanging 500 g weight at 80 °C^52^. Through control of the exposure time of digital light in a single printing run, the SMP actuator has variable mechanical properties in different parts, which allows complex actuation behavior.

In addition, there has been considerable progress in materials research. In 2010, Xie found a tunable multishape memory effect in a perfluorosulfonic acid ionomer (PFSA) belt^26^. After this, Luo et al. developed triple-shaped polymeric composites^38^. Ze et al. reported that a magnetic SMP contains magnetic particles of Fe_3_O_4_ and NdFeB^53^. The Fe_3_O_4_ particles were used for shape locking and unlocking through inductive heating under an alternating current (AC) magnetic field. NdFeB particles provide programmable deformation under a driven magnetic field. Gurevitch et al. showed that by using SMP foams, the artificial muscle can operate with a very low elastic modulus^54^.

Carbon-based shape memory composites have been explored to further improve the performance of artificial muscles. By synthesizing carbon-based conductive shape memory composites^55,56^, thermoelectrical control with Joule heating can be realized conveniently. Moreover, SMP/carbon nanotube (CNT) composites can be driven by the thermal effect of microwaves, although a pure SMP had no response to microwaves^57^. In addition, the mechanical properties can be adjusted using carbon nanomaterials in the composites. Yuan et al. compared polyvinyl alcohol (PVA), PVA-CNT, and PVA-graphene oxide nanocomposite fibers as rotational microactuators^58^. Although the maximum tensile strain was below 30%, high torque with large angles of rotation was achieved when inserting twists into nanocomposite fibers.

### Pneumatic and hydraulic artificial muscles

Pressure-based artificial muscles have been developing rapidly toward soft robotic applications. Equipped with pneumatic and hydraulic artificial muscles, soft robots can provide excellent compliance and safety for interactive applications. Typically, pressure-based artificial muscles can produce forces ranging from 0.01 to 100 MPa, while a typical stroke is 10–100%. The effective modulus of hydraulic artificial muscles is generally higher than that of pneumatic artificial muscles, which means that hydraulic muscles tend to generate higher force. Although conventionally, pressure-based actuators are less preferred in microsystems than other mechanisms such as electroresponsive and thermoresponsive artificial muscles^13^, recent studies have shown that pressure-based micro artificial muscles can also provide excellent force and stroke^59,60^. Some advanced fabrication processes have been studied and have contributed to the miniaturization of pneumatic and hydraulic artificial muscles. For instance, the use of 3D printing can provide extra freedom for their design and fabrication^22,61,62^. Laser cutting can be used for the 2D fabrication of thin pneumatic artificial muscles with high precision^63^. B. Gorissen explored a high-quality reactive ion etching process for PDMS flexible fluidic microactuators^64^. Several systematic reviews of micro pressure-based artificial muscles have been undertaken^13,65^.

Pressure-based artificial muscle has been widely used in emerging soft robots. The boundary between soft robots and artificial muscle research is fuzzy in some cases. These studies usually exhibit some overlap; for example, an artificial muscle can work as an end effector and have a critical influence on the performance of a soft robot. However, the research into soft robotics generally tends to emphasize a systematic architecture; thus, it involves more control strategies and practical applications^66^. Alternatively, artificial muscle research mainly focuses on the innovation and improvement of material and device structure designs^4^.

The structural design has a significant influence on the performance of artificial muscles, such as the strain and produced force. In general, pressure-based artificial muscles consist of elastic chambers and structural constraints. The variation in pressure results in directional deformation. The structural constraints limit the deformation of the actuation to a single dimension to generate extension/contraction, bending, or torsion under control. McKibben muscles with elastic tubes and woven fabric sleeves are classic and have been well studied. The woven and knitted fabrics can also be embedded in the elastomer. These fabrics can stand high pressure up to several hundred kilopascals and produce strains greater than 100%, although the delay and friction loss between the fabric and elastomer is relatively high. Other than fabric-based muscles, pneumatic soft actuators using low-modulus rubbers and multiple air chamber structures can provide compliant and dexterous actuation by controlling the chambers separately, but the produced force is relatively low. Some compliant structures, such as corrugated, folded, and wrinkled structures, can be used with harder materials so that these actuators can achieve both high force output and ultralarge deformation. These actuators have attracted much attention in recent years.

**Fig. 3:**
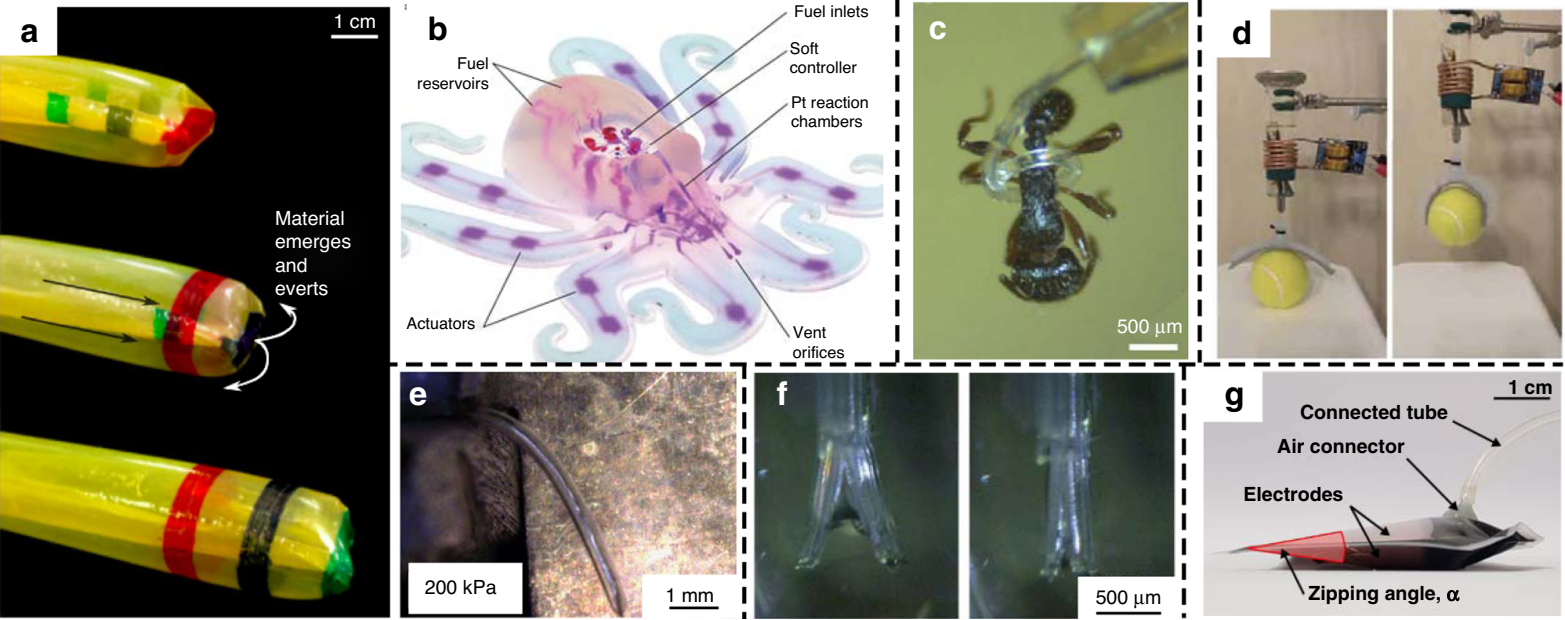
Pneumatic and hydraulic artificial muscles. **a** A soft pneumatic actuator that can extend from the tip^71^. Copyright 2017 by AAAS. Reprinted with permission. **b** A fully soft autonomous robot^69^. Copyright 2016 by Springer Nature. Reprinted with permission. **c** A PDMS micro tentacle with spiral bending capability^60^. Reproduced from ref. ^60^ (2019, CC BY 4.0). **d** An artificial gripper using magnetically induced liquid-to-gas phase transitions^73^. Copyright 2020 by AAAS. Reprinted with permission. **e** A flexible fluidic microactuator^64^. Reproduced from ref. ^64^ (2016, CC BY 4.0). **f** Soft microfingers for cellular aggregate manipulation^75^. Reproduced from ref. ^75^ (2016, CC BY 4.0). **g** Soft electropneumatic pumps^76^. Copyright 2021 by AAAS. Reprinted with permission

The development of soft, small and portable power sources is a major challenge for pneumatic artificial muscles and soft robots^66^. Wehner et al. compared and analyzed several pneumatic energy sources for soft robotics^67^. These authors found that microcompressors had a relatively high capacity but showed low maximum pressure and flow rates. Combustion provided a high energy density, but the peak forces were too high for many soft robots. Cylinders of high-pressure fluid have limited capacity; therefore, the operating time is limited. Monopropellant decomposition (hydrogen peroxide) could provide power with a simple structure but still required system-level development. Nevertheless, some efforts have been made to develop autonomous small soft robots with onboard energy sources^22^. In addition to the power sources mentioned above, electrolysis has also been used to drive diaphragm actuators, as reported by Pang et al.^68^. Figure 3b presents entirely soft, autonomous robots powered by catalytic decomposition of an onboard monopropellant fuel supply^69^.

Pneumatic artificial muscles can generate linear, torsional, and bending actuation, but twisting devices have been less studied than others. Gorissen et al. reported a flexible pneumatic twisting artificial muscle, which provided a bidirectional rotation of 6.5°/mm at a pressure of 178 kPa^70^. In addition, some effective configurations to provide elongation have been explored recently. For example, Hawkes et al. developed a soft pneumatic actuator that can extend from the tip of a body (Fig. 3a)^71^. It has an onboard sensing capability to realize active direction control and is especially useful for navigation in narrow spaces. Blumenschein et al. studied a similar soft tip-extending structure for deploying antennas^72^. Miniaturization is challenging for pressure-based artificial muscles, but many studies have been carried out. Figure 3c shows a micro tentacle that can provide spiral bending actuation and hold an ant^60^. For pressure-based actuators, the produced force is proportional to the actuator cross-sectional area^13^. This means that for microscale artificial muscles, the produced force could be relatively low, but it could be a special opportunity for micromanipulation of some microscale biomaterials (such as devices shown in Fig. 3c, f). Pneumatic artificial muscles have been applied as soft grippers in many studies. For example, Mirvakili et al. presented an artificial pneumatic gripper (Fig. 3d) using magnetically induced liquid-to-gas phase transitions so that bulky peripheral pumps were not needed. The volumetric expansion in the phase transition led to a significant pressure change that can be used for pneumatic artificial muscle^73^. It provided strains of up to 20% within 10 s. Song et al. reported a soft gripper with microfibrillar adhesives on a soft deformable membrane^74^. The soft backing makes the gripper suitable for manipulating parts with complex shapes by actively stretching the soft membrane. Gorissen et al. developed a high-performance etching process for PDMS based on SU8 masks, which were used for flexible fluidic microactuators, as shown in Fig. 3e^64^. Konishi and coauthors developed PDMS-based soft microfingers (Fig. 3f) for pinching and releasing cellular aggregates (200 μm in diameter)^75^ without obvious damage when applying up to 1 mN of restoring force. Diteesawat et al. developed soft electropneumatic pumps employing an electrostatic force and a dielectric fluid, which are only 1.1 mm in thickness and 5.3 g in weight (Fig. 3g)^76^.

### Dielectric elastomer

Electroactive polymers (EAPs) form a class of polymers that can respond to an electric field by generating mechanical motion^77^. These polymers can be simply divided into two categories: field-activated EAPs (such as DEs and piezoelectric polymers) and ionic EAPs (such as IPMC and ionic gels)^9^. In this section, we will first discuss the progress of a DE artificial muscle, which is also known as a DE actuator or DEA.

During the past two decades, DE artificial muscles have attracted increasing attention since R. Pelrine et al. reported the large actuation strain and fast response of acrylic and silicone elastomers in the late 1990s^9,15,78^ because of their fast response, large strains, fracture toughness, and power-to-weight ratios compared to a natural muscle. The basic structure of DE-based artificial muscles can be considered to be a deformable capacitor. This field-actuated pressure across the DE can be simply written as *p* = *ε*_0_*ε*_r_*E*^2,14^, where *ε*_0_ is vacuum permittivity, *ε*_*r*_ is the relative dielectric constant of the DE, and *E* stands for the applied electric field in the DE. Under low strain conditions, the thickness strain *s*_*z*_ can be approximately expressed as *s*_*z*_ = −*ε*_0_*ε*_r_*E*^2^/*Y*, where *Y* is the apparent elastic modulus of the DE.

According to previous research^79^, the viscoelasticity and dielectric strength of the DE artificial muscle need more attention to further improve the actuation performance and prevent it from failing. The viscoelasticity of polymer materials causes significant viscous impedance at high frequencies, which limits the speed of the electromechanical response of DEs. A previous study has shown that the viscoelasticity is induced by the limited rotational freedom of the polymer chains and this viscoelastic loss could be reduced by adding plasticizer into the DE^80^. In addition, the typical driving voltage for the DEA varies from a few hundred to several thousand volts. High voltage leads to high strain, but the maximum voltage is often limited by the electromechanical instability of the elastomer. To decrease the required driving voltage for a required strain level, the dielectric constant could be increased by introducing functional fillers to DEs^81,82^. An example realized both a high dielectric constant and a high breakdown field using conductive polyaniline (PANI) particles encapsulated into an insulating polymer shell before dispersion, as reported by Molberg et al.^83^.

**Fig. 4:**
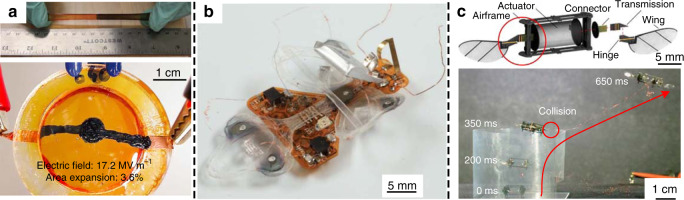
Dielectric elastomer artificial muscles. **a** A highly stretchable self-healing elastomer^84^. Copyright 2016 by Springer Nature. Reprinted with permission. **b** An insect-scale robot driven by low voltage stacked DE artificial muscles^87^. Copyright 2019 by AAAS. Reprinted with permission. **c** Controlled flight of a microrobot using DE artificial muscles^16^. Copyright 2019 by Springer Nature. Reprinted with permission

Many device-level and system-level improvements have been achieved over the last few years. Duduta et al. demonstrated a high-performance soft composite DE artificial muscle design consisting of strain-stiffening elastomers and CNT electrodes. The peak energy density reached 19.8 J/kg^5^. By applying self-healing elastomers, DE devices are able to realize the capability of self-healing^84–86^, which is a feature of natural muscle. Figure 4a shows a self-healing DE artificial muscle reported by Li et al.^84^. The healing process could occur at temperatures as low as −20 °C. Madsen et al. presented a self-healing DE with ionically crosslinked silicone^85^. Two system-level demonstrations are given in Fig. 4b, c. In Fig. 4b, Ji et al. used low voltage stacked DEAs in soft untethered and autonomous legged robots. The insect-scale device can move at 30 mm/s^87^. Figure 4c presents the structure and controlled flight of a microrobot driven by a central DE artificial muscle and related transmission components^16^.

### Ionic polymeric artificial muscle

Another important class of EAPs is ionic EAPs, which work based on the migration of cations and anions in an electric field. IPMCs are typical ionic polymeric artificial muscles consisting of an ion-conducting polyelectrolyte membrane sandwiched between flexible electrodes^13,88^. The polyelectrolyte membrane contains strong ionic groups such as sulfuric acid attached to the backbone of a stable polymer^89^. These highly ionic clusters provide mobile ions. In most cases, anions are fixed on the polymer backbone, while mobile hydrated cations transport to the cathode under an applied electric field, which results in unbalanced stress in the membrane and bending toward the anode direction.

Unlike DE artificial muscles, IPMCs can provide high actuation bending strain in response to a much lower voltage, usually from 1 to 5 V^4,89,90^, although electrolytes with mobile ions are required^90^. The low operating voltage is a significant advantage that allows many biomedical and MEMS applications. However, early IPMCs had to operate in an aqueous environment, so the electrolysis of water molecules and evaporation in open-air significantly limited their performance. Ionic liquids (ILs) have low volatility, wide potential windows, and high ionic conductivity, which can effectively improve the response speed and stability of IPMCs in open air^90^. Another challenge for IPMCs is that the fabrication process of metal electrodes needs to be optimized carefully to balance the flexibility, conductivity, and robustness. The advent of conductive carbon nanomaterials provides a promising alternative option for flexible electrodes^91–93^.

**Fig. 5:**
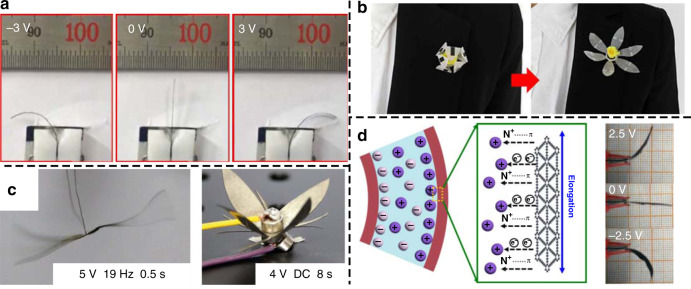
IPMCs and their applications. **a** Low driving voltage IPMC using single-ion conducting polymers^94^. Reproduced from ref. ^94^ (2016, CC BY 4.0). **b** IPMC actuator using MXene electrodes^95^. Copyright 2019 by AAAS. Reprinted with permission. **c** IPMCs are demonstrated to mimic a flapping-wing dragonfly and a flower using AC and DC power sources^97^. Copyright 2019 by Wiley. Reprinted with permission. **d** Graphdiyne-based IPMC actuator with an electromechanical transduction efficiency of up to 6.03%^96^. Reproduced from ref. ^96^ (2018, CC BY 4.0)

Kim et al. reported a high-performance durable IPMC based on single-ion conducting polymers with single-wall CNT electrodes on both sides (Fig. 5a)^94^. This IPMC could provide millimeter-scale displacement under a voltage of only 1 V, while the response time was on the level of tens of milliseconds. This study shows an effective path to improve IPMC artificial muscles by introducing new ionic polymers. In addition, the response times of IPMCs are limited largely by the low mobility of ions at the electrode interface and across the electrode body. To solve this problem, Umrao et al. explored MXene composites as effective flexible electrodes (Fig. 5b)^95^. Their device shows bending strains of 1.37% under sinusoidal input voltages of 1 V and 0.1 Hz. In addition, both of the above two studies realized actuation without back relaxation. Back relaxation is a main disadvantage of IPMCs under DC voltage, which means that the IPMCs will slowly relax after reaching a peak displacement. Figure 5d shows a graphdiyne-based electrochemical actuator^96^ with a high electromechanical transduction efficiency of up to 6.03%. The verified alkene-alkyne complex transition effect is involved in this graphdiyne-based ionic polymeric actuator.

High-performance IPMC artificial muscles have been applied in biomimetic soft robots. As shown in Fig. 5c, Ma et al. reported a Nafion IPMC with homodispersed metal electrodes^97^, which was able to mimic the fast responsive flapping wings of a dragonfly under an AC voltage of 19 Hz, and an artificial flower under a DC voltage. In addition, Must et al. presented an IPMC composed of activated carbon-based electrodes and an IL^98^. With an onboard lithium battery, the untethered biomimetic inchworm could crawl on a smooth surface. Other studies involve applications such as robotic fish^99^.

In addition to IPMC artificial muscles, the improvement of hydrogels in recent years^100,101^ makes ionic artificial muscles based on hydrogels particularly promising because of their outstanding compliance and biocompatibility. Yang and Suo reviewed ionotronic devices, including artificial muscles and electronic skins, based on ionic hydrogels^102^. Carbon nanotubes and graphene can also be used to form ionic artificial muscles^90^. These devices operate based on the electrostatic repulsive force between like charges injected into the actuating nanotube^103^. This kind of actuator often has low strains (<2%) because of the high stiffness of CNTs.

### Piezoelectric artificial muscles

Piezoelectric materials have been widely used in microactuators for a long time. These materials have many applications, such as microlens actuators^104^, rotary^105^ and stepping linear actuators^106^, micropumps^107^, and microgrippers^108^. Previous research has been reviewed extensively in refs. ^109,110^.

**Fig. 6:**
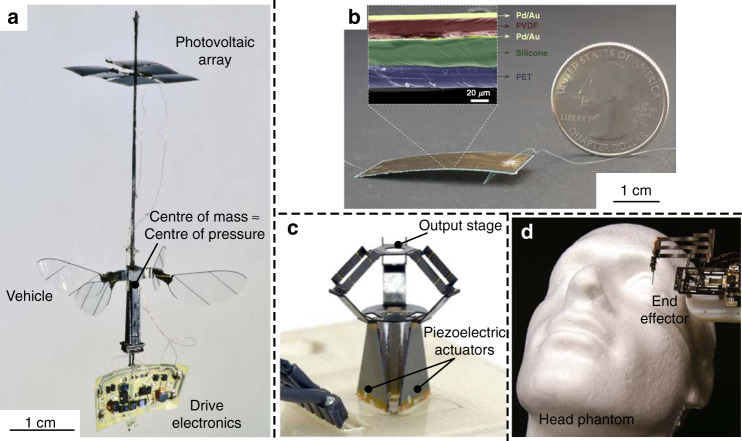
Piezoelectric artificial muscles for microrobotics. **a** An untethered piezoelectric aerial vehicle powered by a photovoltaic array^128^. Copyright 2019 by Springer Nature. Reprinted with permission. **b** Fast-moving soft robot based on PVDF^119^. The motion is in an oscillatory center of mass trajectory pattern similar to a cockroach. Copyright 2019 by AAAS. Reprinted with permission. **c** A millimeter-scale Delta robot driven by three piezoelectric bending actuators^126^. Copyright 2018 by AAAS. Reprinted with permission. **d** Origami-inspired miniature manipulator with a remote center of motion for microsurgery^127^. Copyright 2020 by Springer Nature. Reprinted with permission

Piezoelectric artificial muscles have many advantages, including a simple structure benefiting miniaturization, a relatively high power density at a small scale, and outstanding efficiency. In addition, the improvement of the fabrication process provides extra freedom for the design of micro piezoelectric actuators, such as the 3D printing of piezoelectric materials with designed anisotropy reported by Cui et al.^111^. However, the main challenges for piezoelectric artificial muscles include high stiffness, small strain, and relatively high operating voltage, which are incompatible with artificial muscle applications requiring compliant interaction. The stiffness, or the mechanical impedance, can be decreased by applying soft piezoelectric materials (such as piezoelectric polymers, composites, and other organic piezoelectric materials^112^) and introducing compliant structures^113^. Although soft piezoelectric materials have been widely studied for energy harvesting^114,115^ and sensing^116–118^, at present, there have been fewer studies on actuators based on these materials. Some examples include insect-scale soft robots using curved piezoelectric poly(vinylidene fluoride) (PVDF) films^119,120^. Another cantilever actuator using a PVDF bimorph^121^ was reported by Liu et al. Compliant structures can help to adjust the output strain and force^122^. Vanderborght et al. provided a classification of reported variable stiffness/impedance actuators^123^. Although the actuation strain of piezoelectric actuators is low in general, it can be enlarged by introducing proper amplification mechanisms. For instance, an amplified piezoelectric actuator based on a piezo-hydraulic pump has been developed recently^124^. Kiziroglou et al. recently reviewed strategies and corresponding performances for micromotion amplification^125^. Figure 6c shows a high-precision millimeter-scale Delta robot^126^. The robot is driven by three independent piezoelectric bending actuators and a transmission linkage system. It can be operated in a 7.01 mm^3^ workspace with a high precision of ~5 µm. In Fig. 6d, Suzuki and Woods developed a dexterous origami miniature manipulator with a remote center of motion for minimally invasive surgery, consisting of piezoelectric linear actuators and a pop-up parallelogram structure^127^. The motion range of the end effector could be effectively controlled by changing the leverage structure.

Some system-level progress has been achieved in recent years, as shown in Fig. 6, which suggests the potential toward high-performance autonomous microrobots based on piezoelectric artificial muscles. For example, piezoelectric actuators have been used in flapping-wing microscale aerial vehicles^17,128^. Figure 6a shows an untethered flapping-wing microscale aerial vehicle based on alumina-reinforced PZT actuators powered by a photovoltaic array^128^. The whole system was only 259 mg, and the power consumption was 110–120 mW. This vehicle shows the ability of untethered flight with an additional payload capacity. In addition, an autonomous-legged microrobot with RF communication and an onboard battery was reported by Goldberg et al.^129^. This device realized quick straight-line locomotion at 17.2 cm/s (3.8 body lengths per second). Figure 6b is a biomimetic soft robot with a simple curved configuration based on a PVDF unimorph film^119^, which can run fast with a relative speed of 20 body lengths per second in an oscillatory center of mass trajectory pattern Notably, it could stand the weight of an adult footstep because of the robustness provided by polymeric materials, showing the excellent compliance of these entirely flexible robots.

### Soft magnetic artificial muscles

A magnetic field is effective in providing untethered driving force and control at the same time for microrobots^130,131^. Artificial muscles manipulated by magnetic fields are generally based on discrete or continuous magnetization profiles^13^. Individual micromagnets with proper soft joints are used for discrete magnetization profiles. The continuous magnetization profile requires magnetic particles dispersed across the entire soft polymer^7^, such as PDMS^132^ or hydrogel^130^. These continuous magnetic composites tend to provide better compliance although the magnetization magnitudes are lower^13^.

**Fig. 7:**
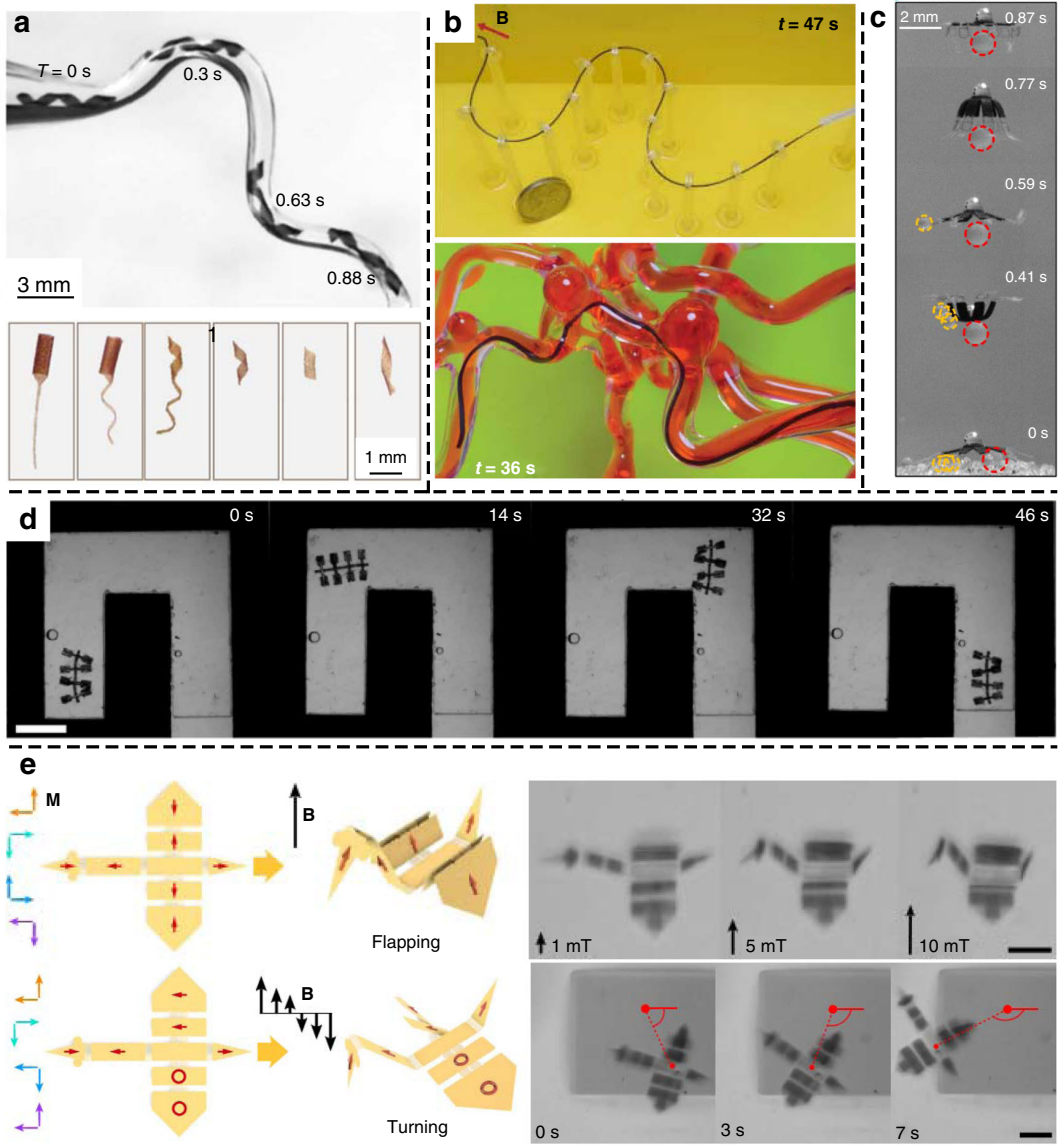
Soft magnetic artificial muscles. **a** Optical images of the engineered artificial microswimmers (upper) and motion of a flexible helix through a curved conduit (lower)^130^. Reproduced from ref. ^130^ (2019, CC BY 4.0). **b** Ferromagnetic soft continuum robots and navigation through a tortuous path (upper) and 3D cerebrovascular phantom network (lower)^132^. Copyright 2019 by AAAS. Reprinted with permission. **c** Micro jellyfish-like swimmer showing the capability of selective transportation of large beads^138^. Reproduced from ref. ^138^ (2019, CC BY 4.0). **d** A flexible robot with programmable three-dimensional magnetization and motion. (scale bar: 4 mm)^139^. Copyright 2019 by AAAS. Reprinted with permission. **e** Microscale origami bird with multiple shape-morphing modes (scale bars: 30 μm)^140^. Copyright 2019 by Springer Nature. Reprinted with permission

As a significant advantage, soft magnetic artificial muscles are usually untethered. The magnetic field brings extra flexibility to this kind of artificial muscle, especially for in vivo operations in the human body. It can penetrate many materials and be spatially programmed^133,134^. An attractive application is microscopic artificial swimmers, which have been developing rapidly in recent years^130,135–137^. These microswimmers use a bioinspired flagellar propulsion mechanism with the construction composed of a microbody and flagellum. As shown in Fig. 7a^130^, Huang et al. reported microswimmers based on a magnetic hydrogel and investigated the adaptive locomotion behavior of microswimmers in a complex fluidic and magnetic environment. In Fig. 7c, Ren et al.^138^. demonstrated a microswimmer that can selectively transport objects in water under the control of an external oscillating magnetic field. Magnetic composite elastomers (Ecoflex/NdFeB) were used as active driving components. Figure 7b presents a study on submillimeter-scale soft continuum robots based on ferromagnetic polymeric composites^132^. The hydrogel layer was used to cover the composites to reduce friction. Figure 7d shows the multilegged paddle crawling of a millimeter-scale soft robot in a microchannel filled with silicone oil. The robot was fabricated based on a newly developed UV lithography method for patterning permanent magnetic particles in a composite film^139^. A micromagnetic robot with a discrete magnetization profile is shown in Fig. 7e^140^. Arrays of programable single-domain nanomagnets on connected panels can be operated in response to the applied magnetic field with high dexterity. The encoded magnetization direction is indicated with red arrows in the diagrams.

In addition to the above six categories of driving mechanisms, artificial muscles can be realized alternatively by molecular machines, chemical fuels (such as glucose^141^), liquid crystal elastomers^142^, and thermoexpansion of polymers and nanocomposites^143^. For example, the development of artificial molecular machines suggests potential possibilities to mimic the natural macromolecular machines in muscles at the molecular level in the future, although current artificial molecular machines are often smaller and simpler than biological macromolecular machines. Particularly, the advent of a stimuli-responsive molecular machine developed by Livoreil et al.^144^ and Bissell et al.^145^ in 1994 has attracted much attention to this field. At the macroscale, some researchers have investigated artificial muscles powered by chemical reactions. Yang et al. developed autonomous crawling robots powered by the catalytic combustion of methanol^146^. Wehner et al. reported an entirely soft robot powered by the gas generated from catalytic decomposition of an onboard fuel^22^. In addition, He et al. reported a novel soft tubular actuator using an electrically controlled liquid crystal elastomer. This actuator provides multiple actuation modes, including contraction, bending, and expansion^142^. Recently, some microsurface electrochemical actuators^147,148^ based on metal films have shown outstanding performance in aqueous environments, for instance, operating at ~1 V and achieving a submicrometer radius of curvature.

Stimulation-responsive gels are another promising option for artificial muscles. This field has developed very quickly in the past decade. Cohen Stuart et al. reviewed stimuli-responsive gels based on the self-assembly of nanoscale structures and materials^149^. Kouwer et al. presented thermoresponsive polyisocyanopeptide hydrogel networks that mimic cytoskeletal proteins^150^. Based on interpenetrating polymer networks, conductive responsive hydrogels have been developed to realize smart hydrogel artificial muscles with sensing functions^151,152^. Zhao et al.^152^ demonstrated a soft somatosensitive actuator using an interpenetrating polymer double-network of poly(N- isopropylacrylamide) (PNIPAAm) and PANI.

## Comparison of electrical and mechanical properties

**Fig. 8:**
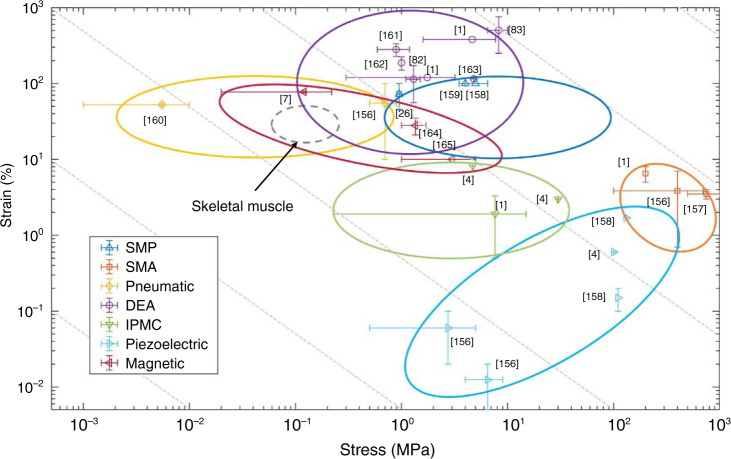
Typical stress and strain characteristics of artificial muscles. These artificial muscles include SMAs^1,156,157^ and SMPs^26,158,159^, pneumatic artificial muscles^156,160^, DEAs^1,82,83,161–163^, IPMCs^1,4^, piezoelectric artificial muscles^4,156,158^, soft magnetic artificial muscles^7,164,165^

The typical stress and strain characteristics of various artificial muscles are shown in Fig. 8, with a skeletal muscle also included for reference. The circles provide the general ranges corresponding to specific mechanisms according to previous reviews^1,7,103,153–155^ and some recent research. The data points show the maximum strain and stress ranges reported in the corresponding references: (a) SMA^1,156,157^ and SMP^26,158,159^; (b) pneumatic artificial muscles^156,160^; (c) DEAs^1,82,83,161–163^; (d) IPMCs^1,4^; (e) piezoelectric artificial muscles^4,156,158^; and (f) soft magnetic artificial muscles^7,164,165^. Among all the artificial muscles, the pneumatic artificial muscles cover the widest range of low-stress actuators. This is a combined effect of material selection, structural design, and power source. Soft magnet-polymer composites have a similar strain range to natural muscle, which suggests the potential to mimic the function and morphology of skeletal muscle. The SMPs can provide a high actuation force and work density with a relatively high strain. Although SMAs have the highest elastic modulus as materials, they are still applied in some soft actuators with low-stiffness structural design^41^.

Among the three kinds of EAPs, DE artificial muscles have the highest maximum strain levels, while IPMCs have a higher elastic modulus than DEs, which suggests that IPMCs can provide higher forces at the same actuation strain. Piezoelectric materials, especially piezoelectric ceramics such as PZT, have the highest stresses but lowest strains among EAPs. This makes piezoelectric actuators suitable for generating fine motion in structures of high mechanical impedance. For piezoelectric actuators, the proper motion amplifying method is required to achieve a larger motion range as discussed previously.

Table 1 presents a further comparison of the parameters of different mechanisms, including the range of maximum actuation strains, elastic moduli, work densities, efficiencies, and relative speeds^1,4,7,12,25,153,156,158^. Artificial muscles based on organic materials tend to have a high actuation strain and a low modulus, similar to natural skeletal muscle, while muscles containing metallic or ceramic components usually have a high elastic modulus. For thermal-responsive shape memory materials, the work densities are attractive; however, the issues of limited speed and low efficiency need to be explored and improved. Pneumatic soft actuators provide medium strain and speed similar to skeletal muscle, while the efficiency varies^23,160,166^. Electrical actuators such as DE and piezoelectric artificial muscles have high efficiency, which is significant for an autonomous system with a limited power supply. In addition, DEs and piezoelectric artificial muscles have high response speeds; therefore, they can be used in applications requiring high-frequency actuation, such as flapping-wing aerial robots^16,128^. The speed of IPMCs is relatively low, but their low operating voltage allows applications that cannot stand the high voltage required by the DE and piezoelectric artificial muscle. Soft magnetic composites have a medium strain level, work density and speed. The reported work density of a millimeter-scale actuator^167^ was relatively low, on the order of 10^−2^ kJ m^−3^, which suggests that the work density of soft magnetic actuators could be limited at small scales. To date, only a limited number of studies have provided the efficiency of magnetic soft actuators. The spatial distribution of the magnetic field could lead to a relatively low efficiency; however, it allows the magnetic artificial muscles to be powered and controlled wirelessly.

**Table 1:** Typical performance of various artificial muscles^1,4,7,12,25,153,156,158^

Mechanism	Actuation strain (%)	Modulus (MPa)	Work density (kJ m^−3^)	Efficiency (%)	Relative speed
SMP	<100	1–1000	<2000	<10	Slow
SMA	<8	10^3^–10^5^	10^4^–10^5^	<10	Slow
Pneumatic	10–100	0.1–100	1–200	<40	Medium
DEA	1–1000	0.1–10	10–5000	60–90	Fast
IPMC	0.5–10	25–2500	1–10	<10	Slow
Piezoelectric	<2	10^3^–10^5^	100–1000	>90	Fast
Soft magnetic	1–100	0.1–10	<300	—	Medium

## Applications of artificial muscles

A large number of applications in various fields have been developed taking advantage of the diversity of artificial muscles. The selection of the actuation mechanism should comprehensively consider the material characteristics and actuation performance, such as the elastic modulus, strength, energy source, efficiency, and response time. Here, we will discuss the adaptability of certain artificial muscles to specific applications, according to the requirements of the application and the properties of the actuation mechanisms.

As shown in Table 2, micro soft swimmers work in liquid environments such as water and blood and thus require extra waterproof properties, especially for electroactive materials. For untethered microswimmers working in the human body, magnetic actuation is preferred^130,137^, which allows wireless control and power transfer at the same time. Hydraulic actuators can be considered for microswimmers to potentially use the surrounding liquid, while it is challenging to develop micro pressure sources for untethered microswimmers. Low-voltage IPMCs with hydrogel materials may also work in water; however, the operating voltage should be below the decomposition voltage of the water.

**Table 2:** Preferred artificial muscles for some typical microsystem applications

Application	Critical requirements	Existing mechanism	Promising mechanism
Microswimmers	Soft structure, waterproof, untethered	Magnetic^130^	Hydraulic, IPMC
Microsurgery	Fine motion, controlled force, quick response, safe	Magnetic^170^, pneumatic^171^, piezoelectric^127^	IPMC
Micro flying robots	High frequency, high power/mass ratio, untethered	Piezoelectric^119^, DE^16^	SMA
Micropumps	Large stroke, quick response, waterproof	Piezoelectric^107^, pneumatic^172^, IPMC^173,174^	Magnetic
Soft grippers	Fine control of force and stroke	Pneumatic^74^, etc.	IPMC
Artificial insect exoskeletons	High power, large stroke, quick response	Piezoelectric^119,120^, IPMC^98^	Pneumatic, DE

Micro soft actuators for invasive surgery need the capability of fine motion, and the force should also be precisely controllable^168,169^. For microsurgery, soft artificial muscle can provide extra compliance and tolerance during invasive treatment. According to the required properties, the potential mechanisms include piezoelectric, magnetic, pneumatic and IPMC actuation. Piezoelectric^127^ and magnetic^170^ actuators can be manipulated with high precision and high speed. Pneumatic artificial muscles have excellent force and locomotion outputs and have be used in microsurgery^171^. However, the response time is relatively long, and the precaution of gas leakage should be considered in some microsurgeries. In addition, IPMCs with low operating voltages provide a safe option, but it is still challenging for an IPMC to provide fine motion compared to a piezoelectric actuator.

Micro flying robots usually need high-frequency actuation up to several hundred hertz, which is challenging for many artificial muscles. To drive the wings of micro flying robots, piezoelectric materials^17^ and DEAs^16^ have been used because of the short response time and acceptable produced force. In addition, micro SMA actuators can potentially be used in this application considering that they have shown the ability to operate above 1 kHz^44^, and the produced force is relatively high. As most SMAs show a one-way shape memory effect, proper structural design is needed to provide periodic movement.

Micropumps can be used in microfluidic devices. The stroke should be large to maximize the driving ability, while the response time should be low to achieve fast control of fluidic flows. A waterproof artificial muscle is suggested considering robustness. Pneumatic actuators^172^ can generate large strain and relatively high force, while piezoelectric actuators^107^ can provide fine control for precise micropumps. IPMCs are able to generate relatively large strokes, and a low operation voltage increases safety^173,174^. Magnetic micropumps are promising with flexible wireless power transmission and control; however, the produced force and power might decline rapidly at the microscale considering the scaling law^13^.

As discussed in previous sections, many mechanisms can be used in soft grippers; here, we provide some examples. Although the requirement for soft grippers varies according to the specific properties of objects and the ambient environment, such as the hardness of materials, chemical components, and ambient temperature, the most basic and general requirement of soft grippers as actuators is the fine control of force and stroke. Pneumatic artificial muscles are most widely explored for this purpose, and many studies have demonstrated their outstanding performance. In addition, soft IPMCs can provide a large stroke using low voltage, and film-based structures of IPMCs need less space in microsystems than pneumatic artificial muscle. However, the relatively low precision and low response speed are considered to be limitations for the application of IPMCs.

Artificial insect exoskeletons can provide the driving force for insect-scale robots, which require actuators with a high energy density, a large stroke and a quick response. Piezoelectric polymers^119,120^ and IPMCs^98^ have been shown to be suitable for this application. An additional advantage of piezoelectric artificial muscles is the high energy conversion efficiency, which allows a longer lifetime for autonomous robotic insects powered by batteries. Micropneumatic actuators are promising and will benefit from studies on pneumatic mechanisms for macroscale robots. The main challenge is the miniaturization of efficient pumps. DEs can provide powerful actuation and a quick response for robotic insects, although the high operating voltage requires relatively complex onboard circuitry^175^.

In addition, scaling laws of various artificial muscles should be considered for designing small-scale devices. Generally, the actuation force and energy are dependent on a characteristic length^176,177^. Diller et al. provided a detailed analysis on this issue^176^. When the scale is below 1 µm, the surface area-to-volume ratio of a microactuator becomes high, and surface-area effects are critical. Viscous forces, friction, and adhesion should not be neglected, and they can be utilized to drive microactuators^12^.

## Energy delivery, storage, and conversion in microsystems

The basic function of the actuation microsystem is to convert energy in various forms into mechanical energy with the required force and motion. Therefore, it is worth considering the most efficient energy management strategies (including energy delivery, storage, and conversion) in this context. Pursuing autonomy is particularly important and challenging for microsystems. Soft robots are typical autonomous systems actuated by artificial muscles. According to Rus and Tolley’s definition, a basic property of soft robots as systems is their capability of autonomous behavior^66^. For autonomous microsystems, untethered energy delivery, with or without local storage, is preferred. In this section, we will focus on energy issues, mainly discussing opportunities in developing energy-autonomous microrobot systems by introducing multistage distributed energy and actuation management.

**Fig. 9:**
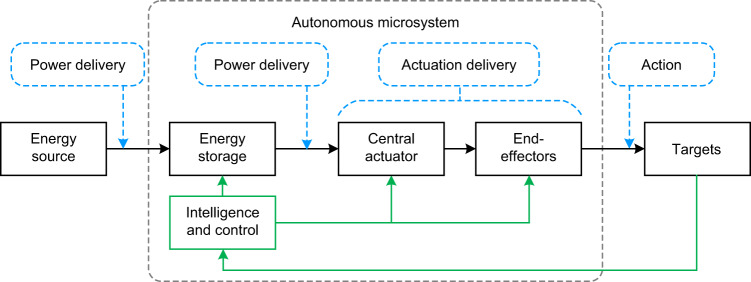
A paradigm of autonomous microsystems enhanced by power and actuation management. Untethered power supplies and proper local energy storage can help microsystems to realize long-term energy autonomy. For microsystems with multiple end-effectors, centralized actuation has the potential to improve the actuating capability and efficiency while minimizing the structural complexity and fabrication difficulty

A paradigm of autonomous microsystems with energy and actuation management can be summarized as shown in Fig. 9. The energy management strategy includes energy storage and delivery, while actuation management involves centralized actuation generation and delivery. The block diagram of Fig. 9 can be thought of as a tool box containing elements that can be repeated and combined in different architectures according to system needs. In the following paragraphs, we will discuss relevant details of these two aspects.

### Energy delivery and storage

A biological example of energy delivery and storage in an autonomous system is the human digestive system and circulatory system. Muscles in the human body consume energy all the time, but people only need to eat food two or three times per day or less to fulfill the daily energy requirement. The energy in the food is taken into the human body intensively in a short time, after which the body uses several stages of energy storage and conversion as “buffers” to make the energy released to tissues slow and smooth. The existence of these energy buffers, such as stomach, liver, and adipose tissue, gives people extraordinary freedom to work and play rather than taking all the time to eat. The above mechanism realizes energy delivery from the environment to the human body and energy storage in the body. Thereafter, the blood in the circulatory system carries nutrients and other essential substances (such as glucose and oxygen) throughout the body. For muscle cells, the blood provides both nourishment and energy, such as glucose, by flowing through the vessels throughout the body to maintain their basic functions and enable their movement under the control of the nervous system. Local chemical energy storage in muscle allows rapid conversion to mechanical effort when the higher output power is needed than can be supplied by the energy delivery rate of the blood.

This biological mechanism is inspiring for the design of autonomous robotic systems. The power and control signals can be delivered in a tethered fashion to the robot body; however, this tethered connection limits the robot’s flexibility. This is especially problematic when the behavior of the robot is complex (such as flying) or when the number of robots becomes large (such as robot swarms). In addition, the low power consumption of the microactuator allows us to choose untethered power supplies with limited power density. Therefore, proper local energy storage and management, as shown in Fig. 9, can coordinate the energy release on demand for actuators in the system temporally and spatially. Rechargeable batteries are the most commonly used energy storage devices for general purposes, while supercapacitors are well suited for applications requiring high power for low duration. By combining one or more of these storage devices with existing wireless power transmission technology^178–180^, the microrobot system can realize long-term energy autonomy. Less explored to date is the wide range of options for centralized, distributed, or localized energy storage. As robotic microsystems become more complex, this design space can be explored to maximize performance within the constraints of space, operating environment, and functional requirements.

In addition to active power delivery, energy harvesting can help by converting ambient energy^181,182^ into electrical power, such as solar cells^183,184^, vibration energy harvesters^185^, stretchable rectennas for RF energy harvesting^186^, biofuel cells^187^, stretchable triboelectric and piezoelectric nanogenerators^114,188,189^. Over the past two decades, major advances in energy harvesting have allowed the emergence of various energy-autonomous devices powered. Micro energy harvesters can be integrated into low-power microactuators or systems. Local storage provision is usually essential in such systems to provide the required energy buffer when demand does not coincide with the availability of supply. In this way, microsystems have opportunities to be entirely energy-autonomous in the future.

### Centralized actuation and actuation delivery

In many scenarios, such as invasive robotic surgery, we can find that several collaborative end effectors could be necessary to complete a task. Conventionally, in these cases, independent driving components are assigned for each end effector. For macroscale or mesoscale robots and actuators, this architecture provides good flexibility for manipulation. However, it causes extra difficulties when the system is scaled down to the microscale. If each of the microactuators requires independent power and actuation arrangements, then the complexity of system design and fabrication will significantly increase.

A promising alternative architecture is centralized multistage actuation. In this architecture, the actuation required by end effectors can be generated by a single high-power central transducer, as shown in Fig. 9. The actuation can be delivered to each end effector in the next stage with a proper transmission mechanism (such as hydraulic, pneumatic, or mechanical linkages such as tendons), so no extra actuating transducer needs to be embedded in these end effectors. Benefiting from centralized actuation, the scale, structural complexity, and fabrication difficulty can be minimized. Moreover, because the central actuator can be placed out of the narrow workspace, it can be designed with more freedom. Compared to the limited scale for an actuator on the end effector, centralized actuation has the potential to improve the maximum actuating capability according to the specific requirement, thus enhancing the performance of the microsystem. Here, macroscale robotic systems also provide inspiration. In macroscale systems, centralized transduction also allows the use of complex, high-efficiency devices (such as hydraulic pumps) while avoiding size constraints and costs or multiple local transducers.

For example, multi-degree-of-freedom microactuators in microsurgery are usually driven by several local actuators; each actuator needs power and control. This makes it hard to decrease the scale of the microactuators. In addition, it can be unsafe when considering the high driving voltage of piezoelectric actuators or the risk of gas leakage of pneumatic actuators. In this case, a central actuator can be used to generate actuation, while a suitable actuation transmission mechanism (such as a hydraulic or leveraged system) can be used to adjust and transfer the required actuation to single or multiple probes at the end. For this architecture, major limitations include material strength and design of the actuation transmission mechanisms, as well as speed limitations and artifacts such as backlash.

## Conclusions

Studies of artificial muscle represent a rapidly growing field. These studies provide innovative approaches for generating actuation with soft materials of outstanding compliance. Here, we have discussed the recent progress in artificial muscle research involving different driving mechanisms, including shape memory materials, pneumatic and hydraulic microactuators, DEs, ionic artificial muscles, piezoelectric microactuators, and micromagnetic artificial muscles.

Each mechanism has pros and cons, so it is suited for different applications. We have discussed preferred driving mechanisms for some typical applications. Output power levels of artificial muscles have been improving in the past decade. Some advanced artificial muscles have already shown competitive power density compared to natural skeletal muscle, but the efficiency of most mechanisms remains at a low level except for the piezoelectric mechanism. The long response time due to the viscoelasticity of polymers limits their use in applications requiring high-frequency movement. Although some studies have been conducted to solve this problem, it is still challenging especially for SMPs and ionic artificial muscles.

We introduce a multistage energy and actuation management paradigm for energy-autonomous microsystems. Flexible energy delivery and local energy storage are promising for improving the reliability and quality of the energy supply and can contribute to the energy autonomy of the microsystem. Centralized actuation generation can potentially improve the overall power efficiency and simplify the design of end effectors in microsystems. Several actuation delivery strategies may be considered for delivering actuation between the central actuator and end effectors. Despite its exploratory nature, this study offers some insight into energy and actuation management for future research and practice.

Artificial muscles are competitive for replacing conventional actuators in areas requiring strong human-machine interaction and strong adaptability to the surrounding environment. In the future, it will be attractive to use this technology for in vivo testing and treatment, drug delivery, micromanipulation of biomaterials, and assistance with physical disabilities. By combining artificial muscle with rapidly developing electronic skin, artificial intelligence algorithms, and wireless power transfer, it is promising for realizing autonomy in many inspiring tasks, such as rehabilitation assistance and swarm intelligence of microrobots in complex environments.
